# Bushfire Smoke in Our Eyes: Community Perceptions and Responses to an Intense Smoke Event in Canberra, Australia

**DOI:** 10.3389/fpubh.2022.793312

**Published:** 2022-02-24

**Authors:** Rebecca Williamson, Cathy Banwell, Alison L. Calear, Christine LaBond, Liana S. Leach, Anna Olsen, Erin I. Walsh, Tehzeeb Zulfiqar, Stewart Sutherland, Christine Phillips

**Affiliations:** ^1^The National Centre for Epidemiology and Population Health, Research School of Population Health, College of Health and Medicine, The Australian National University, Canberra, ACT, Australia; ^2^Centre for Mental Health Research, Research School of Population Health, College of Health and Medicine, The Australian National University, Canberra, ACT, Australia; ^3^Australian National University (ANU) Medical School, College of Health and Medicine, The Australian National University, Canberra, ACT, Australia; ^4^Population Health Exchange (PHXchange), Research School of Population Health, College of Health and Medicine, The Australian National University, Canberra, ACT, Australia

**Keywords:** bushfire smoke, disaster, community, social connectedness, social capital, qualitative

## Abstract

The 2019–20 bushfires that raged in eastern Australia were an overwhelming natural disaster leading to lives lost or upended, and communities destroyed. For almost a month, Canberra, Australia's capital city in the Australian Capital Territory (ACT), was obscured by smoke from fires which threatened the outer suburbs. While smoke itself is experientially different from many natural disasters, it nevertheless poses a significant public health threat. As the impact of extended bushfire smoke in an urban setting is relatively unexplored we aimed to capture the individual and community-level experiences of the event and their importance for community and social functioning. We responded rapidly by conducting semi-structured interviews with a range of Canberra residents who, due to their personal or social circumstances, were potentially vulnerable to the effects of the smoke. Three major themes emerging from the narratives depicted disruption to daily life, physical and psychological effects, and shifting social connectedness. This study highlighted the ambiguous yet impactful nature of a bushfire smoke event, and identified four simple key messages that may be critically relevant to policy making in preparation for similar smoke events in the future.

## Introduction

There is growing consensus that climate change is a significant underlying cause of the extreme bushfire activity witnessed over the “Black Summer” (1–3) of 2019/2020 during which massive bushfires affected much of Eastern Australia. The destruction wrought by the bushfires left widespread devastation for communities directly affected, and millions of people were exposed to unprecedented concentrations of persistent smoke blanketing large parts of eastern Australia. In some places, bushfire smoke impacted air quality for a number of weeks, often in concert with record daytime temperatures (4). Major cities on the east coast of Australia, such as Sydney and Melbourne, experienced record concentrations of air pollution (5). The air pollution was so extensive that it was compared with ratings from some of the most polluted Asian megacities (5). From the perspective of duration and magnitude of smoke exposure, the Hazelwood coal mine fire near Morwell in Victoria in 2014 is the only comparative smoke event in recent Australian history (6). The effects of the smoke were particularly felt in Canberra in the Australian Capital Territory (ACT), where air quality is typically very good (7). On occasions, Canberra's air quality was rated the worst in the world: on New Year's Day, among others, it reached a 24-h PM2.5 concentration of 855.6 μg/m^3^ h concentrations (8) which is well above the WHO standard of 15 μg/m^3^ (9) and the Australian standard of 25 μg/m^3^ (10). Notably, the duration of the bushfire smoke in places like Canberra was extraordinary: from late November to early February 2020 Canberra experienced approximately 40 days when the air quality was considered to be poor (11) including 17 days between 15th December and 15th Februrary when it was extremely poor (≥300 PM_2.5_ μg/m^3^) (12). During this time, the media disseminated health advice to Canberra residents to stay indoors to avoid smoke exposure.

Levels of public concern about the physical health effects of the bushfire smoke were high, particularly for groups at greater risk from smoke exposure, such as those with respiratory or other health conditions (cardiovascular illness, diabetes), older people, young children, and pregnant women (1, 5). This was reflected in significant media attention and public interest in the short- and long-term effects of exposure to bushfire smoke. In early January 2020, the Australian Medical Association (AMA) warned that prolonged exposure to toxic smoke could affect the health of many Australians (13). Recent medical research has demonstrated a range of effects of exposure to bushfire smoke, including the exacerbation of some respiratory conditions, such as asthma and chronic obstructive pulmonary disease, and the onset of acute cardiovascular events in adults (5, 14–16). Two to three years after the Hazelwood fire, self-reported respiratory and poorer psychological health measures symptoms were more common among Morwell participants than comparisons, although there was no increased association between the smoke exposure and cardiovascular disease 4 years later (17). Bushfire smoke has also been associated with lower birth weights in children and increased risk of gestational diabetes in pregnant mothers (18, 19). The recent Royal Commission Report into the 2019/2020 bushfires cited Johnston et al. [(20), p. 42] who that found that:

The 2019–20 season was a major anomaly in the recent record, with smoke-related health costs of AU$1.95 billion. These were driven largely by an estimated 429 smoke-related premature deaths in addition to 3,230 hospital admissions for cardiovascular and respiratory disorders and 1,523 emergency attendances for asthma. The total cost was well above the next highest estimate of AU$566 million in 2002–03 and more than nine times the median annual wildfire associated costs for the previous 19 years of AU$211 million.

The unusual duration and density of smoke over this period prompted the Australian National University to fund studies to assess the effects of this event on the ACT community. At the time of writing, only a few qualitative studies had focused on capturing the individual and community-level experiences of extended bushfire smoke events (compared to the significant literature focused on specifically on fires) or on prolonged smoke events in urban settings. An exception was the 2014 Hazelwood Fire in an open cut coal mine near a regional town. Three years after the fire, the Morwell community remained concerned about lack of planning for similar events and the need to build better community level psychosocial support (21). Qualitative research has also revealed the need for clear consistent public health messaging about the risks of fire and smoke (22, 23).

A substantial body of evidence from disasters around the world consistently recognizes the importance of community and social functioning in disaster response (24). As argued by Kaniasty et al. [(25), p. 337], “individual and collective capacity to triumph over shared adversity is rooted in maintaining and augmenting people's perceptions of being supported and belonging to a cohesive social group and community”. Individual and community social networks provide access to critical resources in disasters and post-disaster situations, such as immediate first response assistance, aid, financial resources and emotional support (24, p 256). Research on individual and community recovery after bushfire/wildfire events demonstrate that most bushfire affected communities may mitigate the negative impacts on health, mental health and wellbeing if they receive adequate social, emotional and institutional support (26–29). In this study, we engage with the concept of social support to explore how people perceived and experienced assistance at the time of the bushfire smoke. Social support—as an overarching concept—is defined as “interpersonal interactions that provide individuals with actual assistance and embed them in a web of social relationships perceived to be loving, caring and swiftly available in a time of need” (23, p. 337). Kaniasty et al. (25) outline three key elements of social support: perceived support, received support and social embeddedness. In practical terms, these three areas translate into helping behavior that might happen (perceived support), helping behavior that did happen (received support), and the network of people who did provide or might provide support (social embeddedness).

The concept of social capital has also been widely applied to understand how communities respond to disasters (30–33). Among many social capital theorists, Putnam [(34), p. 67] has defined it as “the features of social organizations, such as networks, norms and trust that facilitate action and cooperation for mutual benefit”. Through this theoretical lens, social capital points to the webs and networks of support that can ameliorate the effects of disasters and build community resilience (35). Notably, Canberra is considered to have among the highest levels of social capital in Australia according to Australian Federal politician Andrew Leigh, who worked with David Putnam at Harvard (36).

A further consideration is that the societal impacts of natural/environmental disasters are unevenly distributed (37–39), as is exposure to man-made environmental contaminants such as air pollution (40). People impacted by different (and often intersecting) indices of disadvantage, for example, socio-economic status, disability, gender and sexuality, age, residential location, and people suffering from chronic mental and physical health conditions, are likely to bear the brunt of social, environmental and economic devastation caused by natural disasters, and are often more dependent on informal social supports (37). In Australia, Aboriginal and financially disadvantaged groups were more severely impacted by the 2017 Northern New South Wales flood, as they were left with lower social capital than the general community (as indicated through reported informal social connectedness and feelings of trust, belonging, and optimism) (41). This study aimed to respond rapidly to the bushfire smoke by exploring ACT residents' lived experiences of the event, their perceptions of health impacts and the degree to which the smoke impacted their social networks and sources of support.

## Materials and Methods

Canberra, the capital city of Australia, located in the Australian Capital Territory (ACT) has a population of 426,704 (42) people living mainly in the suburbs surrounding five urban centers that make up the city. Reflecting its designation as the Australian bush capital, Canberra is surrounded by farming land, pine forest and heavily wooded and mountainous country. Over a period preceding Christmas and through January, prevailing wind patterns and proximate bushfires in New South Wales meant that Canberra was blanketed by smoke for much of this time (43, 44). At the end of January, a bushfire in a national park in southern ACT came so close to Canberra's southwestern suburbs that residents made evacuation plans and livestock was removed from rural properties. Residents were advised to stay indoors to avoid the smoke and high temperatures. Shortly after the fires finished, some Canberra suburbs were battered by a severe hail storm that destroyed houses and cars. By mid-February 2020, in response to the COVID-19 pandemic, the ACT had restricted interstate travel and then locked down schools and businesses.

The 20 Canberra residents whose experiences inform this study were purposively recruited through researcher networks and snowballing meaning that participants referred the researchers to new potential study participants. The team identified social categories of people that were potentially vulnerable to smoke and social isolation and then actively sought to recruit participants from these groups. Team members contacted neighbors, relatives, work colleagues, community and volunteer organizations. We monitored the sample reflexively to identify gaps. People who had reduced mobility, chronic illness, cared for young children, belonged to a small migrant group, were economically disadvantaged people whose work and hobbies required them to be outdoors for long periods were targeted. Our recruitment strategy is based on our previous successful experience in recruiting marginal and invisible sub-population groups such as people who inject illicit drugs (45) for qualitative research. We intended to conduct sufficient interviews to find patterns across a range of experiences (46) within this small qualitative sample rather than seek population level generalizability.

The interviews, lasting around an hour, were conducted by phone to reduce the risk of COVID-19 transmission. Employing socially distanced research techniques meant that establishing rapport with participants and ensuring a flow of conversation was more difficult (compared to face-to-face interviews), although this approach saved time and travel costs and enabled interviews to be held in a comfortable and familiar environment for both the researcher and interviewee (47). We used a flexibly delivered interview schedule that included topics covering reactions to the smoke and bushfires, methods for managing the bushfire smoke in daily life, perceptions of its impacts on health, key sources of information, and perceptions and experiences of social support and connectedness. The interviews were professionally transcribed and uploaded into QSR NVivo12 for analysis. Members of the research team shared the thematic analysis process (48) by reading interviews, developing a code book and conducting initial coding. Coded text was then sorted inductively, and deductively in response to the study's aims, leading us to generate three major themes, described below.

Ethics approval was given by the ANU Human Research Ethics Committee (approval number: 2020/067).

## Results

We interviewed a total of 20 participants. Our sample was skewed toward women (75%), and older participants: 40% were between 40 and 60 years old, and 40% of participants were aged 60 or over. Table 1 shows how participants were categorized (for some participants several of these categories intersected, e.g., non-English speaking background and parent of young children).

**Table 1:** Participant characteristics.

**Category**	**Number of participants**
Elderly/retired	5
Chronic illness	3
Culturally and linguistically diverse (CALD)	3
Attending drug and alcohol services	2
Parents with young children	3
Outdoor worker/sportsperson	4

Our sample reflected the educational status of Canberra which is higher than the national average (49), 35% of the participants had a Bachelor's degree or above and 35% had a Certificate or Diploma. Forty percent of our sample were not employed or were retired (including those receiving a pension and disability benefit). Four participants (20%) were employed on a part-time or casual basis and four participants (20%) were employed full time. Participants' professional backgrounds varied and included a public servant, childcare worker, surveyor, university tutor, café worker, grounds keeper, vet nurse, and Information Technology (IT) manager. Twenty-five percent of respondents reported renting their homes, while most (75%) were owner-occupiers. Participants were relatively evenly distributed around the Canberra region. Most participants (13, 65%) were married and half had children living with them.

Three major themes emerged from participants' narratives: Disrupting Daily Life, Discomfort and Distress, and Reconfiguring Forms of Community Connection, that covered negligible to highly significant experiences of smoke. We describe each of these in turn, drawing on illustrative quotes from the interviews.

### “Hunker Down, Stay Indoors and Survive”: Disruptions to Daily Life

Participants reported that the bushfire smoke had a significant impact on many aspects of their lives, which required adjustments to their usual routines and practices. They spent less time outdoors than they had prior to the onset of the smoke, which in turn affected regular activities including exercise and hobbies, play, exercising pets, house maintenance, gardening, and social activities. Where possible, participants moved these activities to indoor spaces such as gyms, but occasionally ceased the activity altogether. People were forced to adjust even simple acts, such as whether they opened their house up to improve ventilation. Often people's entire days were scheduled around potential “windows” of less hazardous air quality. These “windows” enabled participants, particularly those with children and animals, to go outside and exercise, walk or play creating a disruption to the temporal patterning of their day.

*So there were, you know, there was the odd sort of morning where I would look out and the sky didn't look too bad. So I would go for a run. (Interviewee #13, male, sportsperson, 40–44 years)*.

Elderly participants, in particular, noted that while the smoke did not stop them from doing most of their everyday activities, their travel, exercise and social activities were reduced or shifted indoors.

*I probably jumped in the car and drove to [nearby suburbs] more often because I could park outside the little supermarket and go straight in and straight out again and that saved me walking for half an hour. So it was like, no walk, get groceries more quickly. But it didn't stop me from driving to visit people. (Interviewee #2, female, elderly/retired, 60*+ *years)*.

However, those with commitments to care for animals or whose employment required outdoor work were more likely to have a more relaxed or stoic attitude toward the smoke, and were less likely to significantly reconfigure their daily routines.

…*there was a need there to keep everything together, and to keep everything working, so you continue regardless. (Interviewee #15, female, outdoor worker, 45–49 years)*.

Families with young children made some of the biggest modifications to routine. December to January in Australia coincides with school holidays and a Christmas close-down period for most workplaces. As a result, a usual weekly routine may not apply in many households. Children often spend summer holidays outdoors, perhaps at the beach, in a pool or in the backyard. While parents reported being concerned predominantly about their children's exposure to the smoke, the extreme summer temperatures also meant that daytime outdoor activities were curtailed. As one participant noted,

*It impacted on any plans we had to visit people, have people visit us. Anything we were going to do before the baby [arrived] was gone. It was just, no, hunker down, stay indoors and survive” (Interviewee #19, female, parent with young child/ren, 40–44 years)*.

Parents explained that children instead embraced “sedentary” activities, leading them to then worry about children's lack of exercise or opportunity to run “amok” outside.

*It was sort of the Christmas that the northern hemisphere must have if they're in, like, Sweden or something. We were inside the whole time. We were doing colouring-in books and writing as if it was wet weather after, you know, day after day. (Interviewee #19, female, parent of young child/ren, 40-44 years)*.

#### Community-Based Outdoor Activities

While heat was also a limiting factor, outdoor events over the summer holiday period (for example, New Year's Eve celebrations, nature reserve open days, sports events) were postponed or canceled due to poor air quality. These events provide a focus for people to come together, socialize and feel a sense of community as this mother explained:

*But certainly over that period of time in summer is often when we're out attending a lot of the smaller community, the outdoor community events. So all the open days at Tidbinbilla [local nature reserve] and all that sort of stuff. We noticed the lack of that very much, because that's sort of our community, I guess. (Interviewee #16, female, parent with young child/ren, 40–44 years)*.

The disruption to organized outdoor activities heightened a sense of abnormality and weakened social ties built by shared scheduled activities.

*So Park Run, if you're familiar with it... [is a] five-kilometre community run on a Saturday morning. I do the one at Belconnen, and, you know, you get your regulars there. And interestingly, they cancelled probably two, I think, of the events, sort of through January, and that was... that was significant in itself. I don't think they'd ever before then cancelled any events for, you know, because of the conditions. So they did that a couple of times…. But it impacted certainly that community quite a lot, I would say (Interviewee #13, male, sportsperson, 40–44 years)*.

In addition, some physical venues, such as outdoor pools, gymnasiums, and outdoor eating areas in Canberra were closed due to the intensity of the smoke. Alternatively, venues were still open but were significantly smoke affected, which made people reluctant to use them:

*And I remember, too, going out to the AIS pool [Australian Institute of Sport, elite athlete training venue that is open to the public], which is a pool that's closed over, and they had all the doors open. And I thought, are you nuts?... and the smell in there was actually worse… (Interview #19, female, parent with young child/ren, 40–44 years)*.

*Yeah, so a lot of the cafés that, you know, that rely on outdoor eating, there was quite a few of them had shut because they couldn't... and again because of the configuration of the café, like, there's one at [upmarket suburb]. It's very, you know, you open everything out and of course, it was very hard to keep the smoke out. A lot of businesses, again, were closing, much like they are now [during COVID]. Some of the braver ones kept going. (Interviewee #12, female, outdoor worker, 60*+ *years)*.

### “Bogged Down and Oppressed”: Discomfort and Distress

The following quotation illustrates the complexities of the impact of smoke on participants' bodies and their emotional states. It also exemplifies the difficulties that people with chronic and other health conditions experienced because they were more susceptible to the physical impacts of breathing in the bushfire smoke. In the following quotation, a woman, who manages multiple complex chronic health conditions, noted her severe reaction to the smoke, which coincided with a change in her medication. Like others, she employed terms such as “concern” or “fear” to describe her response:

*My biggest fear was not being able to breathe, and I did have several asthma attacks. Because I'm an asthmatic, I take medication, but my GP had changed my medication … and I had quite a severe asthma attack…. But I hadn't had an asthma attack since the 2003 fires. (Interviewee #10, female, chronic illness, 60*+ *years)*.

The physical effects of the bushfire smoke included sore eyes and throats, coughing and shortness of breath. Participants reported at least one kind of physical symptom, often more, with symptoms varying across the group.

*I felt I could taste it and I could feel it and I… would get headaches… so I was getting headaches and coughing and feeling terrible… but I think that's the only physical feeling I had but it was, yeah, noticeable (Interviewee #19, female, parent of young child/ren, 40–44 years)*.

*Well, it was sort of hard to breathe and it was... even in our house, inside the house, your eyes were stinging with the smoke inside the house when it was at its worse (Interviewee #20, male, outdoor worker, 50–54 years)*.

The smoke also provoked emotional responses in participants. It was described as: annoying, depressing, oppressive, frightening, disturbing, apocalyptic, surreal, and creepy. One participant observed that the psychological impacts of the smoke derived from multiple factors: changes in routine, an altered lived environment, negative physical health, and reduced exercise and social contact.

…*it was just on top of that psychologically feeling weird and disturbing to have that change in your environment where, you know, people were scarcer, there's lower visibility, it's an oppressive environment, you feel, you know, agitated and things are difficult. It was really affecting on the health, I think, not just for me, but for everybody. And I think it really made people feel bogged down, it oppressed them, it made them feel heavy, they couldn't exercise even in their own homes. I couldn't, not the same and it, like, took away your energy effectively. (Interviewee #11, male, chronic health/accessed drug and alcohol services, 55–59 years)*.

Participants described the severity of the impacts on their mental health as ranging from high levels of anxiety and depression, to feelings of frustration, agitation and despondency. In the following quotations they observed the effects of changes in the physical environment on their emotional states:

*Quite anxious… you know, that's the sky just turning red, you know, anxious and [I] was like, is Armageddon coming? (Interviewee #5, female, non-English speaking background, 30-34 years)*.

*I found the fact that you never saw the sun, you know, day after day, week after week, just that constant, overcast, poor visibility, no sign of blue sky, I found quite depressing. (Interviewee #7, female, chronic illness, 60*+ *years)*.

The physical and mental impact of indoor confinement was as significant for some participants' health and wellbeing as direct smoke exposure. This was strengthened for participants who relied heavily upon the physical and mental benefits of exercise for ameliorating existing mental health conditions:

*My husband, he said at the time, “I have to run because it's worse for me if I don't run…” and he would run even on the worst days, he would go and run. He said there were a couple of times he would get to the top of the hill and he couldn't breathe. (Interviewee #19, female, parent of young child/ren, 40–44 years)*.

Even though the smoke density varied, the duration of the event, from late November to the end of January 2021, was worrying for some.

*The major thing that concerned me, I suppose, was the continuity of it. It just seemed constant and ongoing, and surely it just couldn't keep on going being smoky in Canberra, you know. (Interviewee #11, male, chronic illness/drug and alcohol services, 55-59 years)*.

This led to people feeling that did not have a summer holiday or a break, so that they were not able to replenish their energy and enthusiasm for the new year. One respondent who was a parent described this as a sense of collective depression coupled with anxiety about the bushfire situation.

*I think it just felt like, you know, the whole sort of that Christmas break…got ruined. So you found yourself sort of back at work after not having the normal break you would have for that time of year. And there's a lot of fear and anxiety around, and everyone sort of feeding off each other in that respect. I think it did just feel like everybody was a bit down. Yeah, just a bit flat, and it was really hard to sort of be anything else. (Interviewee #6, female, parent with young child/ren, 30–34 years)*.

Parents of young children spoke about the added pressure of having to manage children's emotions and behavior over this time as children adjusted to restrictions upon outdoor play due to the potential health risks of the smoke. Some sought to manage their children's exposure to media reporting, conversations and images relating to the devastation of the bushfire and smoke events.

*Especially with a four-year-old… And we have a small home and that was difficult for everybody. It was difficult for him, difficult for us, difficult for the baby because she wasn't sleeping as well because her brother kept waking her up. You know, like, it just was... really hard from a mental health perspective. (Interviewee #6, female, parent with young child/ren, 30-34 years)*.

One participant, who worked in a childcare center, noted the emotional impact of the bushfire smoke on children under her care:

*One child is particularly anxious and she says the fire and... she didn't see the fire, obviously, but she seen the smoke in the sky turned dark. She's very kind of wary, anxious. (Interviewee #5, female, CALD parent with young child/ren, 30-34 years)*.

### “We Keep Contact”*:* Recognizing and Reconfiguring Forms of Community Connection

Research has consistently shown that the activation of support networks in times of crisis are critical to mental health outcomes (25). The bushfire smoke impacted on social support and community functioning in many ways. At one level, there was a general decline as organized, outdoor and group-based social activities, including sports events, casual outdoor gatherings, and official events and festivals were canceled or postponed. There was also a reduction in incidental community interactions as people ‘hunkered down' inside their homes and tended to avoid exercising outdoors or using public spaces.

However, many people observed an increase in informal support provided by friends, neighbors and family, and an enhanced sense of social connectedness in the context of a shared crisis. Participants also spoke positively about being in touch more regularly with friends and family over the period of the bushfires and smoke, mainly using social media, video and phone calls:

*Well, we just keep communicating with each other, just check-up everybody is safe. But in terms of support and basically everybody stay inside so not much [socialising in person], but we keep contact with each other. (Interviewee #5, female, CALD parent with young child/ren, 30–34 years)*.

Elderly participants in particular expressed a reliance on immediate family support and regular communication:

*We probably saw them [adult children and grandchildren] twice a week. So Wednesday they'd ring up or we'd ring up and tell them how we're going and things like that. We keep in contact and then if there's anything dramatic happened, we'd ring them straight away. So we were sort of in constant contact. (Interviewee #8, male, elderly/retired, 60*+ *years)*.

However, while immediate support was available, not all participants felt that the severity of the bushfire smoke was fully appreciated by family and friends in other parts of the country or overseas. One female participant reflected,

*Even my family in Sydney and Melbourne, they just were like, “What are you going on about?” …. People didn't get that we were getting that smoke.” (Interviewee #4, female, chronic illness, 50-54 years)*.

Another participant, who was pregnant at the time of the bushfire smoke and had young children, commented:

*My parents were overseas… And I felt that they fully did not understand what we were going through…I mean, they knew they were horrible bushfires, but they just did not understand… how terrifying it was… It just really hurt me that they couldn't understand what it was doing to us all” (Interviewee #19, female, parent with young child/ren, 40-44 years)*.

Material supports either offered or received by participants tended to be based on existing family, friendship and neighborly networks: for example, neighbors checking on each other, family members sourcing air purifiers (which were sold out in local shops and difficult to source) for other each other, or providing accommodation to friends affected by the bushfires. But many participants also offered material support via local community and grassroots groups, for example donating money to bushfire affected communities, or helping to provide food, shelter and water for fire-affected wildlife.

### Support for Vulnerable Groups

For people with chronic health conditions, networks of family, friends, services and neighbors were critical, not only for social support, but also tangible support such as for formulating alternative evacuation plans, dealing with mobility issues, ensuring access to medical services, and coordinating medications.

*Yes, everyone was checking [on me]. And they always do, that whoever's on duty in the fire shed, if they think that I need... if they know that I'm at home, they'll always ring and say did I need to go down to the fire [shed]… if I needed to evacuate quickly... (Interviewee #10, female, chronic illness, 60*+ *years, lives in rural location)*.

Most of the people we spoke to with chronic health concerns felt relatively well supported by friends and family. However, they pointed to significant gaps in support and emergency services that exacerbated their feelings of vulnerability.

*There was this tremendous anxiety about, you know, sure, if my life is in danger a firey [fire rescue worker] will come in and sling me over their back and carry me out. But they're not going to realise that, you know… I'm screaming out for what's going to make me actually able to move around… Everything from, you know, going to the toilet to getting in bed to being able to go for you know, just some fresh air. There was tremendous anxiety in the disabled community about, you know, the step between everything's okay, and this is an emergency, we'll get you out alive. That planning just wasn't possible for people with vulnerabilities. I'm sure that was similar in people who were vulnerable for other reasons… Anyone who didn't have access to a car, anyone who's elderly, anyone who might have mental incapacity, anyone who isn't self-sufficient one hundred percent would have had these issues. (Interviewee #4, female, chronic illness, 50-54 years)*.

Financially vulnerable people were also at risk from social isolation. One participant did not use social media, phone apps or television much but he did have a mobile phone. He observed that getting out and exercising, despite the smoke, was necessary for his mental health and sense of social connectedness:

*I've got a lot of experience with depression and anxiety which makes me very shy and kind of socially isolates me. So, you know, getting out to me is really important - to kind of exercise as well, you know, like, just to refresh me mentally… it's just super important to me (Interviewee #11, male, chronic illness/drug and alcohol services, 55-59 years)*.

He felt that being able to volunteer in some way can also be significant in times of crisis, to enhance a sense of utility and belonging to networks of mutual support. He had previously noted that he “found caring about other people helped me to be less concerned about myself”, and reflected:

*In the early days… I wanted to volunteer and I looked for ways to enlist and volunteer and help out any way I could. Even if it's just putting blankets on people, you know, just to help out, just to take the load off someone else. I found it difficult for a couple of reasons... I was looking online for ways to volunteer and help, and the phone numbers that I tried were, like, you know, just don't lead you anywhere helpful…. But what disappointed me was there seemed to be no appeal to help utilise, you know, people's willingness to help… (Interviewee #11, male, chronic illness/drug and alcohol services, 55-59 years)*.

However, he was unable to action these plans, due to poor communication from services. Participants also sourced support and information from community groups based on, for example, mutual interests or ethnic identity. This support was mainly coordinated through social media and community group webpages. Examples included members of a local multiple sclerosis support group circulating information about smoke exposure to members, an advice page created by the local horse riding community to navigate animal care in response to the smoke and bushfires, and a religious group checking-in on its members and distributing face masks:

*Well, we [members of her religious group] just keep communicating with each other, just check-up everybody is safe. But in terms of support and basically everybody stay inside so not much, but we keep contact with each other…. So our group just shared for everybody a, like, emergency list… like a checklist. So whatever the things you need to prepare, just getting things ready if the fire gets close. (Interviewee #5, female, CALD/parent with young child/ren, 30-34 years)*.

Recent migrants to Canberra also relied on culturally specific social support networks, most of which were existing groups communicating via social media. One respondent commented:

*The Indonesian community in Canberra are pretty solid. And we had the WhatsApp group, the social media who are offering help and support, too (Interviewee #17, female, CALD/parent with young child/ren, 45-49 years)*.

For people who live alone or are at risk of social isolation, particularly if they have pre-existing mental illness, social supports during such crises are critical, particularly where the inability to participate in the public sphere or access usual services are curtailed (50). Several participants felt this kind of support was lacking over the smoke event. In addition to creating “alert lists” and better outreach communication, one participant suggested:

*Maybe you could use public libraries… where they have clean air, to invite these people to be enclosed in the regular business side of the library for a moment. But we're loathe to do that when we come across a disaster…. So that would have to be a predetermined decision to do something like that under extreme conditions and consider, people that, you know, whatever conditions they have: mental, physical, what kind of impairment they have, to try and be embracing, comforting, and help them on the path to better health. So enabling these people would be a good thing… how we can use people's ability to contribute, how we can provide a safe environment for them and inclusion (Interviewee #11, male, chronic illness/drug and alcohol services, 55-59 years)*.

### Heightened Solidarity

Participants observed negative emotions such as anxiety and fear within their local community. At the same time, people also commented on a heightened sense of solidarity and camaraderie based on shared experiences and hardship. For some participants this changed their usual interactions with neighbors and also created new opportunities for meaningful, albeit transitory, interactions with strangers:

*I remember, you know, on that horrible Sunday [a day of peak invisibility with an Air Quality Index of over 3000], I had to go out for my daily coffee…. And I remember on that Sunday when it was just so soupy and there was a little café… and … there was a few brave people out having coffee, and just a bit of camaraderie and, you know, I think I sat there for about an hour talking to absolute strangers. So I think it all brings you together… I think there's a bit of bonding. You know, you're all going through the same situation, some worse than others. I think you see a bit more kindness in people. (Interviewee #12, female, outdoor worker, 60*+ *years)*.

Another female participant spoke about connecting with neighbors in one of Canberra's southern suburbs (which were on high alert from a bushfire in the south of the ACT), where families “*who probably hadn't spoken to each other before in their own neighbourhoods”* gathered at in a public reserve to watch the fire front and share information.

Several of the participants focused on the altruistic responses from the community, which created a broader sense of (both local and national) collective identity and belonging:

“…*we've all got that human tendency to pull together when we have to. And a lot of people did…. the good human traits can come out in adversity, I guess. And that's something we definitely saw through the fires, and I particularly felt very proud to be an Australian and to be, you know, in a community where there were such capable people doing as much as they possibly could to try and help. (Interviewee #16, female, parent with young child/ren, 40-44 years)*.

Many Canberra residents have a close connection to the South-east New South Wales coast, where they own beach houses, spend holidays or where family and friends reside, and community solidarity was extended to some of the worst-hit bushfire zones on the coast:

*I think there's, you know, [in] any sort of difficult situation is a feeling of togetherness as well, that you're all sort of… you're all facing the same sort of ordeal. And I feel like there was a sense of community, not just in the Canberra region but sort of extending down to the coast as well because obviously Canberrans like to get down there… (Interviewee #13, male, sportsperson/parent with young child/ren, 40-44 years)*.

## Discussion

Participants' narratives revealed disruptions to their everyday routines as they experienced physical discomfort and mental distress. Their social networks were temporarily reconfigured when they turned to nearby neighbors, family and friends for support when outdoor and other community group activities were interrupted by dense smoke. Close proximate social ties rapidly filled the gap in organizational connections and with distant family. People “pulled together”, neighbors checked-in on each other, and some face-to-face communications were replaced by phones and digital media. More formal connections, based on outdoor social, sporting and work activities were halted while people remained indoors and “hunkered down” to avoid the intense smoke, the longevity of which meant that people felt that social life had been placed “on hold” (51). Canberra residents may have been particularly strongly affected by the smoke, as they have high levels of involvement in outdoor activities (52) due to easy access to green space, and normally good quality air. Indeed, the presence of smoke—as a continued interference to everyday interactions in the natural environment—was significant for almost everyone we spoke to: parents struggled to entertain children inside, elderly members of the community were unable to take their daily walk, outdoor workers were unable to work, and other people reliant on outdoor exercise for their mental and physical wellbeing were impacted.

Disaster literature is often concerned with immediate life threatening events (floods, fires, and earthquakes) focusing on “survivors”, “post-traumatic stress”, and the destruction of physical assets (25). While smoke, and heat to a lesser extent, were less immediately life-threatening in Canberra, the event fulfills the definition of a disaster as a “basic disruption of social context within which individuals and groups function” [(53), p. 651 cited in (25)]. It shared similarities with man-made air pollution in other countries (54, 55) and with drought which produces an increased risk of poorer physical and mental health (56) but less obvious threat to mortality.

Social relationships are considered key to resilience, given that “the most essential, and the most reliably present, characteristic of all disasters is that they exert a strong impact on social relationships” (23, p. 344). Kaniasty et al.'s (25) qualitative and quantitate scoping review of disaster studies in Australia and Oceania finds that social support from family, friends and community is important for psychological wellbeing. Kaniasty et al. (25) also found that the nature and strength of support may become attenuated over time. Given that the Canberra smoke event lasted about 6 weeks, the disruption to community services and activities was minor, but could be problematic for vulnerable or disadvantaged people if extreme fire, heat, and smoke events return for longer periods.

These findings aligns with studies that show that people living in close proximity who normally interact little, reach out in times of difficulty potentially forming longer term social connections (25). This adaptive response may have been further strengthened during the ensuing COVID-19 pandemic and lockdowns. A qualitative study on community connectedness after the Christchurch earthquakes (51) observed that strong pre-existing community connections aided recovery. Often, public social events are useful in counteracting deteriorating social support after disasters (57), but the poor air quality prevented these occurring in Canberra and then later during the COVID-19 outbreak which occurred soon after. In conceptual terms, social support was immediately useful to understand how social relationships act as a resource for individuals when confronting life stressors. Participants more readily spoke about, and relied upon, expressions of emotional, material and informational support from friends and family, and were less likely to speak about the structural supports and wider civic group membership that are emphasized by social capital scholars such as Putnam and Coleman (35). These are more likely to be disrupted during periods of bushfire smoke. Being embedded in social networks was important to our participants' experiences. However, we concur with Saegert and Carpiano (35) that the concept of social support should be integrated into social capital theory to better understand social context, structural elements, and community level phenomena, as other Australian studies have done [see (58)].

Canberra, ACT is considered to have among the highest levels of social capital in Australia (36). Explanations for this include its medium sized population, time freed up by short commutes, access to shared outdoor spaces and a comparatively homogeneous, well-educated, well-resourced population (36). An earlier study of Australian social capital found that Canberra residents had high capacity to raise money, participate in the labor force, and be active group members (59). Compared to rural or remote localities that have experienced disasters (60), the resources and social networks characteristic of living in a medium-sized city, such as Canberra, may ameliorate social isolation and post-disaster recovery, as this study found. Nevertheless, marginalized groups may still be excluded from the benefits of social capital (61). Most participants in this study accessed networks, information and support although, those with financial hardship and mental health problems, had greater difficulties. The study points to the value of funding and supporting civil society organizations so that they may be employed in future emerging disasters, and particularly events of this duration.

As noted above consistent clear messaging about health risks is important (22, 23). From analysis of the interviews we identified four very simple key messages: (1) *To communicate the health impacts of smoke clearly*. Even though most participants experienced physical and mental discomfort and ill health from smoke, they were uncertain about how serious a threat the smoke was to their health, particularly for vulnerable adults and children, and how to protect themselves from exposure. (2) *To expect and prepare for poorer psychosocial health*. Most people found that their mood deteriorated while sheltering from smoke—sometimes in unexpected ways. (4) *To assume that organized social connections and support will be disrupted*. Even though Canberra residents generally enjoy high levels of social capital, it is likely that they will need to rely on more proximal social support during periods of crisis. Building and maintaining connections with proximate social contacts will ameliorate disconnection in future events. (6) *The government could do more to assist those who are disadvantaged and vulnerable by improving resources, social infrastructure, and communication*. Participants expressed the view that disadvantaged ACT residents should not have to rely on their own limited resources and networks to access masks or air purifiers. They suggested that government should take action to protect against future similar events including measures to identify, track and support vulnerable and socially isolated people and to provide easy access to services and equipment for the duration. Similarly, experts have noted that government advice usually focusses on the immediate aftermath of a disaster does not plan for long term events such as this (62). During the subsequent COVID-19 lockdown in the ACT, vulnerable people registered for support and receive deliveries of groceries and medications—a system that could be reactivated for other disasters.

### Study Strengths and Limitations

Overall our sample provided a range of perspectives on the bushfire smoke and reflects some characteristics of the Canberra population who tend to be relatively well-resourced and educated. Within this context, the sample was purposively recruited from groups who may have been somewhat more reliant on social networks and social connectedness for assistance: those managing chronic health conditions, people accessing drug and alcohol services or at risk of social isolation, people caring for young children, people belonging to smaller culturally and linguistically diverse groups, elderly people, or people working outdoors. The study aimed to respond rapidly to the smoke event, even though ethnographic qualitative research has traditionally required long periods of fieldwork. However, the Canberra-based researchers had existing local knowledge. The interviews which were conducted within 3 months of the event reduced the risk of retrospective memory biases. The use of phone interviews expedited data collection but potentially reduced rapport with some participants while enabling self-disclosure for others (63). As noted, this study drew upon concepts such as social support and social capital to interpret the impact of the smoke on social relationships, and the degree to which the risk of social isolation was ameliorated through social ties. Generally, relationships between social capital and inequality are measured to determine the impact of environmental and other crises on affected communities (30). However, this approach was not appropriate in a small exploratory qualitative study such as this. The study responds to calls for more in-depth qualitative research in this area to understand the contextual and cultural factors that shape the needs of vulnerable groups and to improve community adaptive capacity and disaster resilience (41). Finally, the experiences and memories of some participants were partially overtaken by a severe hailstorm on January 20, 2020 that resulted in extensive damage then followed by the emerging COVID-19 pandemic. Participants in our studies on social connectedness and COVID-19 (forthcoming) noted the cumulative and exhausting effect of these multiple disasters, highlighting the need for systemic research approaches that recognize the interconnections between health and environmental disasters.

## Conclusion

While, in general, participants reported considerable disruption to their daily routines, and psychological and physical discomfort and distress, they indicated relatively high levels of perceived support, received support and social embeddedness (25). However, significant gaps were identified that highlight opportunities for building community resilience for future bushfire smoke events. Furthermore, social inequalities that exist in the city were somewhat exacerbated by the smoke event. While we have pointed to the strength and resilience of Canberra's residents in the face of the smoke, heat and fear of fire, it is important to recognize that people found it an unsettling and anxious time, particularly in the context of expectations that these types of events are likely to occur more frequently due to climate change. Lessons learnt from this event may help allay people's fears of similar events in the future and can be shared with other communities.

## Data Availability Statement

The datasets presented in this article are not readily available because in compliance with ethics advice, interview transcripts are not available. Requests to access the datasets should be directed to cathy.banwell@anu.edu.au.

## Ethics Statement

Ethics approval was given by the ANU Human Research Ethics Committee (approval number: 2020/067). The patients/participants provided their written informed consent to participate in this study.

## Author Contributions

CB, AC, CL, LL, AO, CP, EW, and SS: project design. RW: data collection. TZ and EW: literature review. CB and RW: data analysis. CB and RW: manuscript draft. AC, EW, CL, LL, CP, AO, and SS: critical revision of the article. All authors contributed to the article and approved the submitted version.

## Funding

This work was supported by a seed funding grant from the College of Health and Medicine (ANU). AC was supported by a National Health and Medical Research Council (NHMRC) Fellowship 1173146.

## Conflict of Interest

The authors declare that the research was conducted in the absence of any commercial or financial relationships that could be construed as a potential conflict of interest.

## Publisher's Note

All claims expressed in this article are solely those of the authors and do not necessarily represent those of their affiliated organizations, or those of the publisher, the editors and the reviewers. Any product that may be evaluated in this article, or claim that may be made by its manufacturer, is not guaranteed or endorsed by the publisher.

## References

[1] Commonwealth of Australia. Royal Commission into National Natural Disaster Arrangements Report. (2020).

[2] Lockie S. Sociological responses to the bushfire and climate crises. Environ Sociol. (2020) 6:1–5. 10.1080/23251042.2020.1726640

[3] Vardoulakis S Marks G Abramson MJ. Lessons learned from the australian bushfires: climate change, air pollution, and public health. JAMA Intern Med. (2020) 180:635–6. 10.1001/jamainternmed.2020.0703 32108854

[4] Ulpiani G Ranzi G Santamouris M. Experimental evidence of the multiple microclimatic impacts of bushfires in affected urban areas: the case of Sydney during the 2019/2020 Australian season. Environ Res Commun. (2020) 2:065005. 10.1088/2515-7620/ab9e1a

[5] Vardoulakis S Jalaludin BB Morgan GG Hanigan IC Johnston FH. Bushfire smoke: urgent need for a national health protection strategy. Med J Austral. (2020) 212:349–53.e41. 10.5694/mja2.50511 32088929 PMC7318141

[6] Johnson AL Dipnall JF Dennekamp M Williamson GJ Gao CX Carroll MT. Fine particulate matter exposure and medication dispensing during and after a coal mine fire: a time series analysis from the Hazelwood Health Study. Environ Pollut. (2019) 246:1027–35. 10.1016/j.envpol.2018.12.085 31159135

[7] ACT Health. Air Quality in the ACT. (2020). Available online at: https://www.health.act.gov.au/about-our-health-system/population-health/environmental-monitoring/monitoring-and-regulating-air

[8] La A Patel M Hunter A Phillips C. Towards resilient health systems for a more extreme climate: insights from the 2019/20 Australian bushfire season. Int J Wildland Fire. (2021) 30:1–5. 10.1071/WF20083

[9] WHO. WHO Air Quality Guidelines. (2021).

[10] Australia. National Air Quality Standards. Ambient Air Quality. (2016). Available online at: https://soe.environment.gov.au/theme/ambient-air-quality/topic/2016/national-air-quality-standards

[11] Brown A,. Canberra Air Quality: More Than A Third of All Summer Days Had Hazardous Air Quality. The Canberra Times (2020). Retrieved from: https://www.canberratimes.com.au/story/6665438/just-how-bad-was-the-air-quality-in-canberra-this-summer/

[12] Rodney R Swaminathan A Calear A Christemsen B Lal A Leviston Z. Physical and mental health effects of bushfire and smoke in the Australian Capital Territory 2019-20. Front Public Health. (2021) 9:682402. 10.3389/fpubh.2021.682402 34722432 PMC8551801

[13] Australian Medical Association. AMA Warns of New Health Threats From Ongoing National Bushfire Crisis. (2020). Retrieved from: https://ama.com.au/media/new-health-threats-escalating-bushfire-crisis.

[14] Leibel S Nguyen M Brick W Parker J Ilango S Aguilera R. Increase in pediatric respiratory visits associated with santa ana wind-driven wildfire smoke and PM2. 5 Levels in San Diego County. Ann Am Thorac Soc. (2020) 17:313–20. 10.1513/AnnalsATS.201902-150OC 31860802

[15] Walter C Schneider E Knibbs LD Irving LB. Health impacts of bushfire smoke exposure in Australia. Respirology. (2020) 25:495–501. 10.1111/resp.13798 32180295

[16] Cascio WE. Wildland fire smoke and human health. Sci Tot Environ. (2018) 624:586–95. 10.1016/j.scitotenv.2017.12.086 29272827 PMC6697173

[17] Ikin J Carrol T Walker J Borg B Brown D Cope M. Cohort profie: the hazelwood health study adult cohort. Int J Epidemiol. (2020) 49:1777–8. 10.1093/ije/dyaa083 32728690

[18] Holstius DM Reid CE Jesdale BM Morello-Frosch R. Birth weight following pregnancy during the 2003 Southern California wildfires. Environ Health Perspect. (2012) 120:1340–5. 10.1289/ehp.1104515 22645279 PMC3440113

[19] Melody SM Ford JB Wills K Venn A Johnston FH. Maternal exposure to fine particulate matter from a large coal mine fire is associated with gestational diabetes mellitus: a prospective cohort study. Environ Res. (2020) 183:108956. 10.1016/j.envres.2019.108956 31831154

[20] Johnston FH Borchers-Arriagada N Morgan GG. Unprecedented health costs of smoke-related PM2.5 from the 2019-20 Australian megafires. Nat Sustain. (2021) 4:42–7. 10.1038/s41893-020-00610-5

[21] Yell S, Duffy, M, Whyte, S, Walker, L, Carroll, M, Walker, J,. Hazelwood Health Study. Community Wellbeing Report Volume 2: Community Perceptions of the Effectiveness of Community Rebuilding Activities. (2019). Available online at: https://hazelwoodhealthstudy.org.au/__data/assets/pdf_file/0009/2059236/CW-Report-Volume-2_version-1.0.pdf

[22] Marfori M Campbell SL Garvey K McKeown S Veitch M Wheeler A. Public health messaging during extreme smoke events: are we hitting the mark? Front Public Health. (2020) 8:465. 10.3389/fpubh.2020.00465 32984250 PMC7492534

[23] Fish J Peters MD Ramsey I Sharplin G Corsini N Eckert M. Effectiveness of public health messaging and communication channels during smoke events: a rapid systematic review. J Environ Manage. (2017) 193:247–56. 10.1016/j.jenvman.2017.02.012 28226261

[24] Quarantelli EL Dynes RR. Response to social crisis and disaster. Annu Rev Sociol. (1977) 3:23–49. 10.1146/annurev.so.03.080177.000323

[25] Kaniasty K de Terte I Guilaran J Bennett S. A scoping review of post-disaster social support investigations conducted after disasters that struck the Australia and Oceania continent. Disasters. (2020) 44:336–66. 10.1111/disa.12390 31298760

[26] Camilleri PJ Healy C Macdonald EM Nicholls S Sykes J Winkworth G. Recovery from bushfires: the experience of the 2003 Canberra bushfires three years after'. J Emerg Primary Health.(2010) 8:1–15. 10.33151/ajp.8.1.112

[27] Bryant RA Waters E Gibbs L Gallagher HC Pattison P Lusher D. Psychological outcomes following the Victorian Black Saturday bushfires. Austral N Z J Psychiatry.(2014) 48:634–43. 10.1177/0004867414534476 24852323

[28] Berger E Carroll M Maybery D. Children's perspectives on the impact of the Hazelwood mine fire and subsequent smoke event. PsyArXiv. (2018) 3. 10.31234/osf.io/8mhxf

[29] Botey AP Kulig JC. Family functioning following wildfires: Recovering from the 2011 Slave Lake fires. J Child Fam Stud. (2014) 23:1471–83. 10.1007/s10826-013-9802-6

[30] Aldrich DP Meyer MA. Social capital and community resilience. Am Behav Sci. (2015) 59:254–69. 10.1177/0002764214550299

[31] Dynes RR,. The Importance of Social Capital in Disaster Response. University of Delaware Disaster Research Center Preliminary Paper #327 (2002). Retrieved from: https://udspace.udel.edu/bitstream/handle/19716/292/PP%20327.pdf.

[32] Elliott JR Haney TJ Sams-Abiodun P. Limits to social capital: Comparing network assistance in two New Orleans neighborhoods devastated by Hurricane Katrina. Sociol Q. (2010) 51:624–48. 10.1111/j.1533-8525.2010.01186.x 20939128

[33] Hawkins RL Maurer K. Bonding, bridging and linking: How social capital operated in New Orleans following Hurricane Katrina. Br J Soc Work. (2010) 40:1777–93. 10.1093/bjsw/bcp087

[34] Putnam RD. Tuning in, tuning out: The strange disappearance of social capital in America. Polit Sci Polit. (1995) 28:664–84. 10.2307/420517

[35] Saegert S Carpiano RM. Social support and social capital: a theoretical synthesis using community psychology and community sociology approaches. In: Bond MA Serrano-García I Keys CB Shinn M editors. APA Handbook of Community Psychology: Theoretical Foundations, Core Concepts, and Emerging Challenges. American Psychological Association (2017). p. 295–314. 10.1037/14953-014

[36] Davies A,. Why Does Canberra Have More Social Capital. Crikey Inquiry. (2010). Available online at: https://blogs.crikey.com.au/theurbanist/2010/10/13/why-does-canberra-have-more-social-capital/

[37] Klinenberg E. Denaturalizing disaster: a social autopsy of the 1995 Chicago heat wave. Theory Soc. (1999) 28:239–95. 10.1023/A:1006995507723

[38] Parkinson D. Gender and disaster: literature review. In: Australia: Women's Health Goulburn. Wangaratta, VIC: North East Inc. (2011).

[39] Veenstra G. Social capital and health (plus wealth, income inequality and regional health governance). Soc Sci Med. (2002) 54:849–68. 10.1016/S0277-9536(01)00049-1 11996020

[40] Graham S. Life support: the political ecology of urban air. City. (2015) 19:192–215. 10.1080/13604813.2015.1014710

[41] Matthews V Longman J Bennett-Levy J Braddon M Passey M Bailie R. Belonging and inclusivity make a resilient future for all: a cross-sectional analysis of post-flood social capital in a diverse Australian Rural Community. Int J Environ Res Public Health.(2020) 17:7676. 10.3390/ijerph17207676 33096716 PMC7589110

[42] ABS. Regional Population. Australian Bureau of Statistics (2019). Available online at: https://www.abs.gov.au/statistics/people/population/regional-population/latest-release,

[43] Khaykin S Legras B Bucci S Sellitto P Isaksen L Tence F. The 2019/20 Australian wildfires generated a persistent smoke-charged vortex rising up to 35 km altitude. Commun Earth Environ. (2020) 1:1–12. 10.1038/s43247-020-00022-5

[44] Mannheim M. NSW fires will continue to blanket Canberra for foreseeable future, experts say. (2020). Available online at: https://www.abc.net.au/news/2020-01-08/why-canberra-is-a-smoke-bowl/11845592

[45] Olsen A Banwell C Dance P. Internal or infernal devices: experiences of contraception among Australian women living with hepatitis C. Health Care Women Int. (2009) 30:456–74. 10.1080/07399330902797591 19418320

[46] Saunders B Sim J Kingstone T Baker S Waterfield J Bartlam B. Saturation in qualitative research: exploring its conceptualization and operationalization. Qual Quant. (2018) 52:1893–907. 10.1007/s11135-017-0574-8 29937585 PMC5993836

[47] Irvine A. Duration, dominance and depth in telephone and face-to-face interviews: a comparative exploration. Int J Qual Methods. (2011) 10:202–20. 10.1177/160940691101000302

[48] Braun V Clarke V. What can “thematic analysis” offer health and wellbeing researchers? Int J Qual Stud Health Wellbeing. (2014) 9. 10.3402/qhw.v9.26152 PMC4201665 25326092

[49] ABS. Census Quickstats. Australian Bureau of Statistics (2016). Available online at: https://quickstats.censusdata.abs.gov.au/census_services/getproduct/census/2016/quickstat/CED801

[50] Zakour MJ Harrell EB. Access to disaster services: Social work interventions for vulnerable populations. J Soc Serv Res. (2004) 30:27–54. 10.1300/J079v30n02_0333314144

[51] Thornley L Ball J Signal L Lawson-Te Aho K Rawson E. Building community resilience: learning from the Canterbury earthquakes. Kotuitui N Z J Soc Sci. (2015) 10:23–35. 10.1080/1177083X.2014.934846

[52] SportAus. Australia's Most Active States and Territories Revealed. (2019). Available online at: https://www.sportaus.gov.au/media_centre/news/australias-most-active-states-and-territories-revealed

[53] Fritz CE. Disasters. In: Merton RK Nisbet RA editors. Contemporary Social Problems. New York, NY: Harcourt (1961). 651–94.

[54] LeVan TD Koh WP Lee HP Koh D Yu MC London SJ. Vapor, dust, and smoke exposure in relation to adult-onset asthma and chronic respiratory symptoms: the Singapore Chinese Health Study. Am J Epidemiol. (2006) 163:1118–28. 10.1093/aje/kwj144 16707657 PMC1509764

[55] Zhou Y Liu J. Air pollution and mental health of older adults in China. Sustainability. (2020) 12:950. 10.3390/su12030950

[56] Edwards B Gray M Hunter B. The social and economic impacts of drought. Austral J Soc Issues. (2019) 54:22–31. 10.1002/ajs4.52

[57] Bourke JA Hay-Smith EJC Snell DL Schluter PJ. Community inclusion of wheelchair users during the long-term recovery phase following the 2010/2011 Canterbury earthquakes. Int J Disaster Risk Reduct .(2017) 23:169–77. 10.1016/j.ijdrr.2017.05.004

[58] Berry HL Welsh JA. Social capital and health in Australia: an overview from the household, income and labour dynamics in Australia survey. Soc Sci Med. (2010) 70:588–96. 10.1016/j.socscimed.2009.10.012 19931236

[59] Bureau of Transport and Regional Economics. Focus on regions No. 4 Social Capital. Information Paper Dept. of Transport and Regional Services (2005).

[60] Alston M. '“It's really not easy to get help”: services to drought-affected families'. Austral Soc Work. (2007) 60:421–35. 10.1080/03124070701671149

[61] Villalonga-Olives E Kawachi I. The dark side of social capital: a systematic review of the negative health effects of social capital. Soc Sci Med. (2017) 194:105–27. 10.1016/j.socscimed.2017.10.020 29100136

[62] Cowie CT Wheeler AJ Tripovich JS Porta-Cubas A Dennekamp M Vardoulakis S. Policy implications for protecting health from the hazards of fire smoke a panel discussion report from the workshop landscape fire smoke: protecting health in an era of escalating fire risk. Int J Environ Res Public Health. (2021) 18:5702. 10.3390/ijerph18115702 34073399 PMC8199356

[63] Trier-Bieniek A. Framing the telephone interview as a participant-centred tool for qualitative research: a methodological discussion. Qual Res. (2012) 12:630–44. 10.1177/1468794112439005

